# Smokeless Tobacco Pouch Keratosis: A Report of Two Cases

**DOI:** 10.7759/cureus.116283

**Published:** 2026-09-15

**Authors:** Ramachandra Reddy Gowda Venkatesha, Karthik Rajaram Mohan

**Affiliations:** 1 Oral Medicine and Radiology, Vinayaka Mission's Sankarachariyar Dental College, Vinayaka Mission's Research Foundation (DU), Salem, IND

**Keywords:** buccal vestibule, smokeless tobacco (st), smoking cessation clinics, tobacco cessation, tobacco pouch keratosis

## Abstract

Tobacco pouch keratosis (TPK) is a white lesion caused by chronic contact with smokeless tobacco (SLT) on the oral mucosa. The lesion develops as a result of chronic tobacco chewing or snuff dipping. The lesion exhibits precancerous characteristics; thus, dentists must educate patients about the dangers of tobacco use and advise them to quit. This article discusses the clinical findings of a 17-year-old adolescent boy and a 48-year-old man who were SLE users diagnosed with TPK and nicotine replacement therapy to averse from the SLT chewing habit due to nicotine dependence. The novelty of this study was that TPK typically manifests clinically as a thick, whitish wrinkled appearance in areas specifically extending along the vestibule or gingiva in contact with SLT and these clinical insights of SLT pouch keratosis underline the importance of early detection and the critical role of dental professionals in managing SLT-related lesions.

## Introduction

Smokeless tobacco (SLT) refers to a variety of tobacco products that are consumed without burning. These products are used by placing them in the mouth or nasal cavity, where the nicotine is absorbed through the mucous membranes. A lack of knowledge about the dangers of health and peer use are all factors that contribute to the use of SLT [1]. SLT presents a complex public health challenge. While it doesn't involve inhaling smoke, it still carries significant health risks, particularly with long-term use. Understanding its types, use patterns, and health implications is crucial for developing effective public health strategies and interventions aimed at reducing tobacco-related harm [1]. The prevalence of tobacco pouch keratosis in Chengalpattu district, Tamil Nadu was 5% [2].

SLT used in South India

Pan masala, Hans, betel quid, and Gutkha are the types of SLT most commonly used in South India among the several variations [1]. Paan, often referred to as Betel Quid, is a mixture of areca nut and betel leaf as its primary ingredients. One of the nouns used in several South Asian languages is called paan, which literally means "leaf." The betel leaf is made up of several different components that are contained within it. Generally, paan comprises tobacco, slaked lime, spices, quenched lime, and areca nut wrapped in betel quid [1].

Pan masala

A mixture of processed tobacco, betel leaves, rose petal paste that has been sweetened, sugar, clove, cardamom, coriander seeds, herbs, dried dates, and amla is used to prepare pan masala [1].

Gutkha

Areca nuts (the dry fruit of Areca catechu), powdered tobacco, and slaked lime (including aqueous calcium hydroxide) are the primary components that go into the production of gutka, which is a kind of SLT. The gutkha is made from slaked lime, spices, catechu areca nuts, and chewing tobacco [1]. It is typically packaged in a bag or tin. Not only does the presence of high-quality areca nut grains in gutka cause mechanical irritation to oral tissues, but it also makes it possible for ground tobacco to attach to the injured mucosa, which results in morphological changes and damage to the membranes. As a consequence of this, the consumption of areca nuts in conjunction with tobacco can speed up the development of oral submucous fibrosis in individuals who consume gutka of a consistent nature [1].

This case report aims to identify SLT pouch keratosis based on the characteristic clinical findings of a whitish wrinkled appearance that develops in contact areas of the SLT and avoid invasive biopsy procedures in diagnosing such lesions.

## Case presentation

Case one

A 17-year-old adolescent boy reported to our Department of Oral Medicine and Radiology in Ariyanur, Salem, Tamil Nadu for a routine dental checkup. There was no associated medical history. History revealed that he had the habit of chewing SLT in the form of Hans and pouched it in his mandibular anterior labial vestibule five times a day for the past three years. Intraoral hard tissue examination revealed dark brownish extrinsic stains near the cervical third on the labial aspect of the crown portion of both the right and left mandibular central incisors. On further closer soft tissue examination, a whitish wrinkled appearance was present on his labial aspect of attached gingiva concerning his right and left mandibular central incisors, mandibular labial sulcus, mandibular anterior labial vestibule, and labial mucosa, which are in frequent contact with the SLT (Hans) (Figure 1).

**Figure 1:**
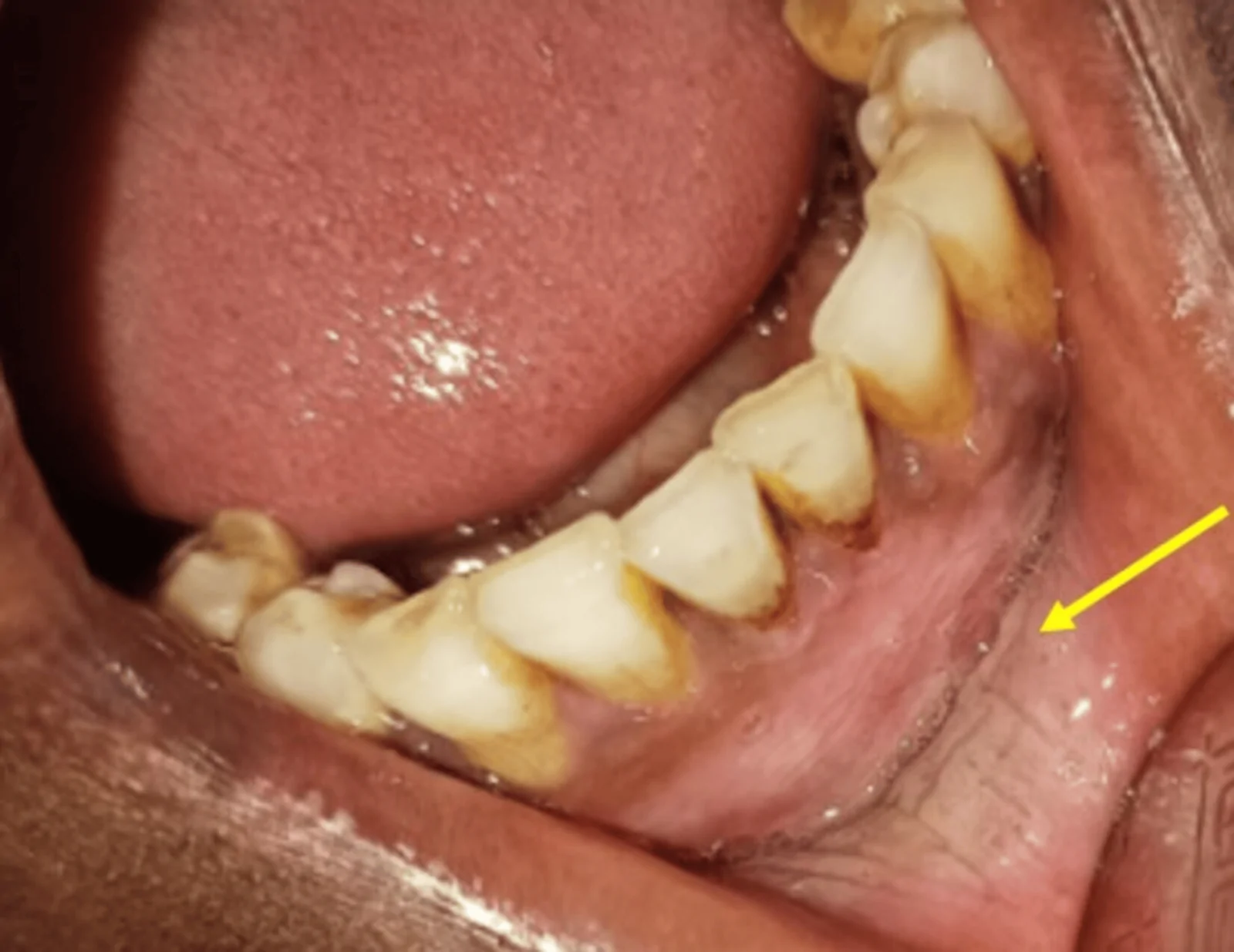
Case one: Intraoral examination revealed a whitish wrinkled appearance involving mandibular labial vestibule (yellow arrow)

On palpation, it was rough, leathery in texture, non-scrapable, and non-tender. Correlating the history of pouching the SLT frequently in the mandibular anterior buccal vestibule and the onset of the lesion, it was provisionally diagnosed as tobacco pouch keratosis (TPK). The differential diagnosis includes SLT-induced leukoplakia. The SLT-induced leukoplakia lesions are differentiated by the presence of white homogeneous or non-homogeneous white plaque and the absence of wrinkled appearance. The SLT pouch keratosis is characterized by a white or gray-white wrinkled appearance commonly involving the contact areas of the SLT with the oral mucosa.

Case two

A 48-year-old male reported to our Department of Oral Medicine and Radiology in Ariyanur, Salem, Tamil Nadu for a routine dental checkup. His medical history was not hypertensive, asthmatic, or any other systemic illness. The patient is not under any other medication for any other systemic illness. History revealed a habit of pouching SLT in the form of Hans in his mandibular anterior labial vestibule. A general examination revealed that his vitals were stable. Intraoral hard tissue examination revealed brownish-black extrinsic stains along the cervical third of the crown portion of mandibular right and left central and lateral incisors. Soft tissue examination showed blackish SLT particles near the mandibular labial vestibule concerning the right mandibular central incisor region. Further closer examination also revealed a whitish wrinkled appearance involving the contact areas of SLT, namely the mandibular anterior labial vestibule and labial mucosa, which are in contact with the SLT in the form of Hans (Figure 2). On palpation, it was rough in texture, leathery in consistency, and non-scrapable.

**Figure 2:**
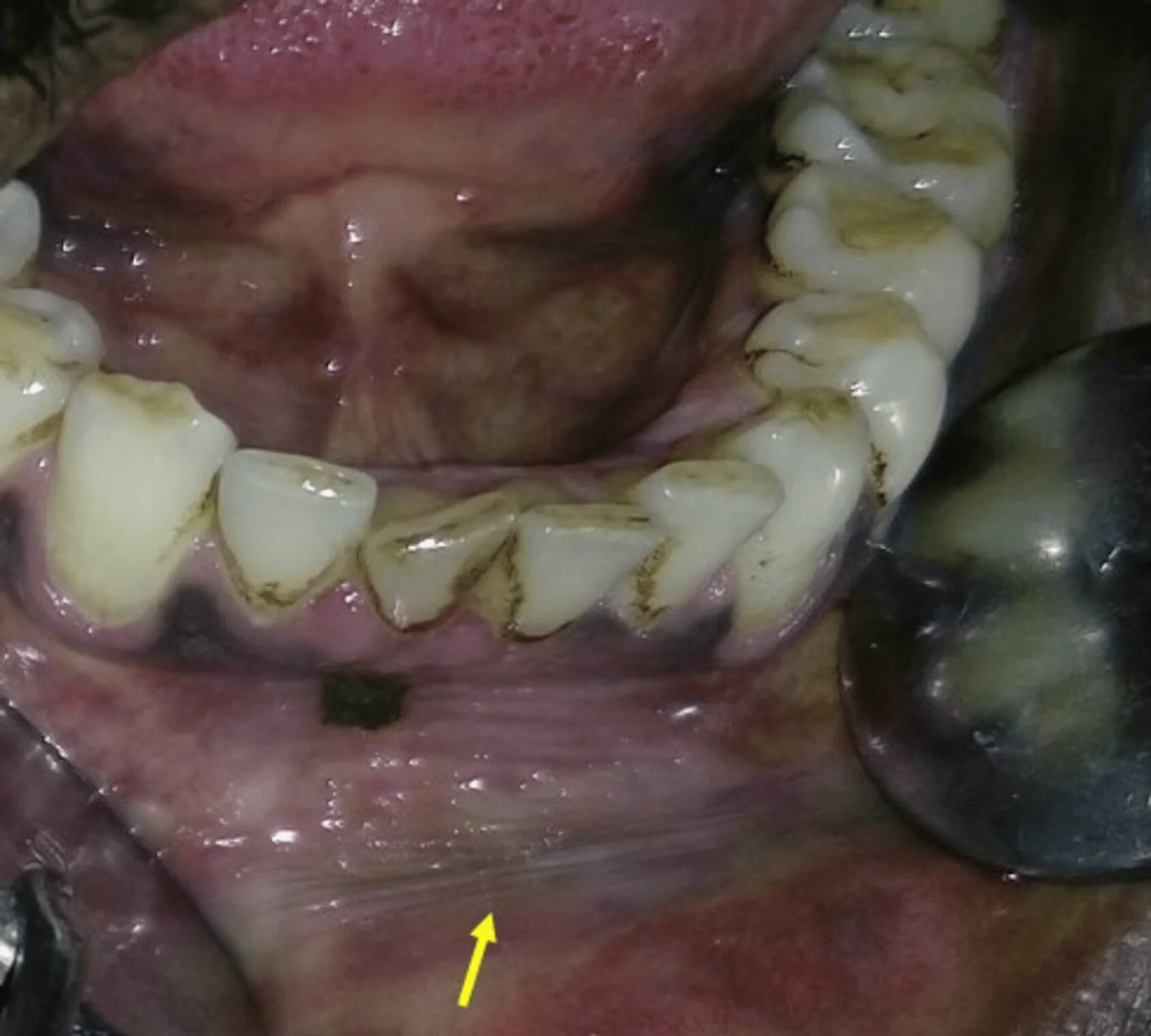
Case Two: Intraoral examination revealed tobacco-induced extrinsic stains and a whitish wrinkled appearance involving the mandibular anterior labial vestibule in relation to mandibular central and lateral incisor region (yellow arrow)

Nicotine replacement therapy was suggested in both of the above cases. SLT chewers were advised to start with 4 mg of nicotine Polacrilex (Nicorette®) chewing gum. The patients were asked to chew one piece of nicotine gum every 1-2 hours at first or whenever they had the urge to chew SLT. The patients were asked to slowly chew the chewing gum for a minute and place (park) it in their buccal vestibule. The patients were then asked to reduce nicotine chewing gum intake by one piece every 4-7 days and chewing time per piece from 30 to 10-15 minutes for 4-7 days. After seven days, the patients were asked to chew 2 mg nicotine gum instead of 4 mg gum and follow the steps mentioned above. The patients were advised to avoid using not more than 24 pieces of 2mg or 12 pieces of 4mg nicotine chewing gum per day for more than three months. The patients were followed after a month. The urge to chew and pouch SLT significantly diminished, but the lesion persisted in both the above cases.

The clinical significance of this case report is that TPK is a clinical sign that indicates the habit of pouching SLT. The occurrence of TPK in an adolescent male indicates that this condition is a prevalent health issue among young individuals. Dentists must be aware of identifying such lesions during screening of the oral cavity and provide nicotine replacement therapy to averse from the harmful habit of pouching tobacco due to nicotine dependence.

## Discussion

Several carcinogens are found in SLT, with tobacco-specific nitrosamines being the primary agent responsible for the development of cancer. Nitrosamino acids, polycyclic aromatic hydrocarbons, and aldehydes are other carcinogens in SLT [3,4]. The nicotine in SLT is the cause of keratosis of the oral epithelium resulting in a whitish wrinkled appearance [4]. The anterior mandibular vestibule region is the most prevalent location for observing TPK, followed by the posterior mandibular vestibule and maxillary labial vestibule where it is rarely observed. As the condition develops, the lesion increases in thickness and wrinkles and becomes more rigid and difficult to move against. Scientific investigations have proven that the smoking of tobacco during adolescence can interfere with brain circuits and have harmful effects on cognitive capacities, leading to attention deficiencies and psychiatric diseases [4].

Epidemiology

The calculated chronic tobacco usage rate among males was 54.1%, whereas in females it was 15% [5]. This study examined 96 people from north Maharashtra, India, who used SLT and developed unilateral SLT pouch keratosis. Information on demographics, historical and current use of SLT, characteristics of SLT pouch keratosis, dentition status index, modified community periodontal index, and loss of tooth attachment were all gathered for the study. According to the findings of the study, there was a noteworthy distinction (p≤0.05) in gingival bleeding, attachment loss between teeth and pocket depth that had SLT keratosis, and teeth that were located on opposing sides of the dental arch respectively. There is a substantial relationship between the length of time spent using tobacco and the degree of attachment loss at the sides of the SLT pouch keratosis. When comparing the SLT pouch keratosis and contralateral sides, there was a significant difference (p≤0.05) in the average evaluations for sound, healthy non-carious crown, carious crown, coronal caries, and their status. The study found that SLT pouch keratosis is related to severe gingival bleeding, gingival recession (GR), and loss of attachment in the teeth compared to teeth on the contralateral side without lesions [6].

The incidence of GR was 87.5%. Haathichaap was the predominant form of SLT used, accounting for 35.9% of users, closely followed by the nonpackaged kind (loose tobacco) at 19.4%. Similarly, these patients' periodontal parameters were seen to a greater extent in a descending sequence [6]. The GR and SLT placement are substantially correlated in this study. This cross-sectional investigation shows that TPK abnormalities increase periodontal disease risk. Raising awareness of periodontal risks, oral cancer, and their health effects can enhance patients' oral and overall health and quality of life [6].

A thorough study was performed on 345 patients diagnosed with TPK in Mangalore, Karnataka. All of these patients were recognized as SLT users. The patients had clinical examinations to assess numerous clinical periodontal indicators, including GR, extrinsic stains, increase in periodontal pocket resulting in mobility due to furcation involvement, and the effect of various kinds of SLT on periodontal health in TPK regions [7]. The clinical examinations that were performed on the patients were designed to evaluate a variety of clinical periodontal parameters, including stains, GR, periodontal pocket, furcation involvement, mobility, and the local effects of various forms of SLT on periodontal health in TPK regions [7]. The study included 5,000 participants, 1,085 (21.7%) used tobacco. 273 (25.2%) smoked tobacco, and 811 (74.7%) used SLT. The most prevalent lesion was TPK with 46.1% [8].

Among the 5,613 patients, there were 1,537 cases of TPK [8]. A total of 1000 individuals were involved in this cross-sectional study-individuals with a propensity for tobacco consumption. TPK was the most prevalent lesion identified in those who chew tobacco [9]. Candida species other than Candida albicans, present in leukoplakia and TPK, are likely to be prevalent concerning saliva production rate and buffering capacity. Because of this, antifungal treatment choices need to respond quickly to resistance and the change in prevalence from Candida albicans to Candida species other than albicans [10]. Tobacco use causes apparent dentition changes as well as latent modifications in the oral mucosa. Two out of every 100 individuals studied had TPK [11]. The use of SLT was primarily observed among younger boys. Lesions particular to certain habits, such as TPK, tobacco-induced lichenoid reactions, and oral submucous fibrosis, were observed in individuals who use SLT [12]. Among the 80 samples, 30 were diagnosed as leukoplakia, whereas 45 were identified as TPK [13]. Among the 50 individuals tested, there was a prevalence of 30% for TPK [14]. Among the 880 tribes in Tamil Nadu, the prevalence of leukoplakia and TPK in the entire study population was 1.6% [15]. Among the 3200 individuals examined in the eastern development zone of Nepal for oral cancer and potentially malignant oral illnesses, TPK was the most prevalent condition, accounting for 50.4% of cases [16]. A cross-sectional study conducted in Guntur City, Andhra Pradesh, South India, found that 42.4% of the 300 participants had TPK and other soft-tissue lesions [17]. TPK was identified in 34.2% of participants in the age category of 25-44 and 31.4% of those in the age group of 45-60, according to the findings of a cross-sectional study that was carried out among 488 sevayats of the Shree Jagannath Temple in Puri [18].

Clinical features

The presence of nicotine in saliva provides an ideal environment for Candida's continued multiplication and expansion. Therefore, smoke causes an elevation in the concentration of Candida. The tobacco-induced changes in the mucous membrane may be attributed to a vicious cycle of alterations. This process entails increased levels of pro-Candida chemicals due to the modified acidic pH of saliva in individuals who chew tobacco. Consequently, there is increased growth of Candida, which is associated with compromised defense mechanisms [19].

Treatment

Nicotine replacement therapy is indicated for TPK to averse from the SLT pouching habit due to nicotine dependence. TPK superimposed with candidiasis is treated effectively with curcumin. Curcumin demonstrates antifungal properties by causing the breakdown of the fungal candidal plasma membrane. The researchers discovered that the depletion of cytoplasmic potassium results in the demise of fungal cells [19]. Curcumin-induced acidification of the cells reduced the outflow of H+ and triggered the death of Candida [19]. Furthermore, curcumin hindered the growth of hyphae by explicitly targeting the global suppressor thymidine uptake 1 (TUP1)c [19].

## Conclusions

TPK is a persistent, prevailing health problem, creating a burden in society due to the growing number of SLT users from adolescents to adults. It is crucial to manage SLT pouch keratosis due to nicotine addiction through nicotine replacement therapy and the cessation of SLT use. The involvement of healthcare experts in promptly recognizing SLT-related diseases is essential, and patients should be informed about the detrimental implications linked to the persistent use of SLT. 
